# Shrinkage Optimization in Talc- and Glass-Fiber-Reinforced Polypropylene Composites

**DOI:** 10.3390/ma12050764

**Published:** 2019-03-06

**Authors:** Youngjae Ryu, Joo Seong Sohn, Byung Chul Kweon, Sung Woon Cha

**Affiliations:** Department of Mechanical Engineering, Yonsei University, Seoul 03722, Korea; yjryu1027@yonsei.ac.kr (Y.R.); ssamjjang87@yonsei.ac.kr (J.S.S.); kwonb@yonsei.ac.kr (B.C.K.)

**Keywords:** shrinkage, talc, glass fiber, polypropylene, injection molding, ANOVA, the Taguchi method, regression analysis

## Abstract

The shrinkage of reinforced polymer composites in injection molding varies, depending on the properties of the reinforcing agent. Therefore, the study of optimal reinforcement conditions, to minimize shrinkage when talc and glass fibers (GF) (which are commonly used as reinforcements) are incorporated into polypropylene (PP), is required. In this study, we investigated the effect of reinforcement factors, such as reinforcement type, reinforcement content, and reinforcement particle size, on the shrinkage, and optimized these factors to minimize the shrinkage of the PP composites. We measured the shrinkage of injection-molded samples, and, based on the measured values, the optimal conditions were obtained through analysis of variance (ANOVA), the Taguchi method, and regression analysis. It was found that reinforcement type had the largest influence on shrinkage among the three factors, followed by reinforcement content. In contrast, the reinforcement size was not significant, compared to the other two factors. If the reinforcement size was set as an uncontrollable factor, the optimum condition for minimizing directional shrinkage was the incorporation of 20 wt % GF and that for differential shrinkage was the incorporation of 20 wt % talc. In addition, a shrinkage prediction method was proposed, in which two reinforcing agents were incorporated into PP, for the optimization of various dependent variables. The results of this study are expected to provide answers about which reinforcement agent should be selected and incorporated to minimize the shrinkage of PP composites.

## 1. Introduction

Injection molding is used in the production of complex plastic products [1,2,3]. In the case of plastics, in this process, molten resin is injected into a mold and is then left to cool and solidify into the final plastic product. During this process, the cooling in mold causes shrinkage, and the product is reduced in size, compared to the mold dimensions. This phenomenon occurs as a result of the difference in the cooling rate at the surface of the product and that of the interior [4]. If an injection-molded part experiences a large direction-dependent shrinkage deviation, its dimensional stability is seriously affected. This might also cause warpage in this part. Therefore, various studies have been conducted to reduce such shrinkage and warpage problems [5,6,7,8,9].

Zafar et al. applied a microcellular foaming process to reduce linear and volumetric shrinkage of the injection-molded parts [5]. The authors used acetal copolymer as a material, and by applying the foaming process, they were able to reduce the shrinkage and weight of the injection-molded samples. Jin et al. carried out a finite element analysis to predict the residual stress and distortion in smartphone baseplates manufactured by die-casting and injection-molding processes [6]. The results of the finite element analyses were compared with the actual experimental values, and it was found that the thickness of the plate caused an uneven residual stress, which, in turn, created local distortions. Bensingh et al. analyzed the volumetric shrinkage and deflection in an injection-molded Bi-aspheric lens, using a computer numerical simulation [7]. Polycarbonate was used as the material, and the optimal injection-molding process conditions to minimize volumetric shrinkage were identified. In addition, experiments with the optimal process parameters were carried out, and it was found that the injection-molded Bi-aspheric lens had a shallow and steep surface profile accuracy.

Direction-dependent shrinkage deviation was more varied, when a reinforcing agent was incorporated into a polymer, thus forming a polymer composite, compared to that of the polymer alone. In particular, a reinforcing agent with a high aspect ratio exhibits a large shrinkage deviation, according to the direction in which it aligns with the flow of the polymer [10]. As a result, shrinkage in the direction parallel to that of the polymer flow (that is, the flow direction (FD)) is different from that in the direction perpendicular to that of polymer flow (that is, the transverse direction (TD)). Depending on the reinforcement type, polymer composites exhibit different shrinkage tendencies, so the choice of reinforcement is very important. Therefore, many studies have been conducted to confirm the shrinkage of reinforced polymers [11,12,13,14,15,16]. For example, Juraeva et al. predicted the mechanical properties and volumetric shrinkage of injection-molded automobile components [12]. They investigated various polymer composites compounded from six types of base materials (polyoxymethylene, polyamide, polyphthalamide, polyphenylene, and polyetherimide) and two types of reinforcements (glass and carbon fibers), which were simulated to obtain the optimum volumetric shrinkage, tensile strength, and flexural strength, among other factors. In addition, Cadena-Perez et al. measured the shrinkage and warpage of the glass-fiber-reinforced polypropylene (PP), as a function of the compatibilizers used in the injection molding [13]. They confirmed that the shrinkage and warpage were reduced by increasing the content of the compatibilizing agents.

Many studies have been carried out on the shrinkage of polymer composites containing only one reinforcing agent to date, but there is a lack of research into the differences in the shrinkage of reinforced polymer composites, depending on the type of reinforcement used in injection molding. Therefore, in this study, we aimed to investigate how shrinkage changes with the reinforcement type, reinforcement content, and reinforcement size, when talc and glass fiber (GF) are incorporated with the base material, PP. Talc and GF are commonly used reinforcements in industry and are worthy of analysis. PP is worthy of analysis as a semi-crystalline polymer resin that has a thermal behavior that is different from that of amorphous polymers [17,18,19,20,21]. We compared the variation in the shrinkage trends at different locations in the injection-molded parts. This is because the shrinkage tendencies of talc- and GF-reinforced PP composites, in different regions, have not been extensively studied. In addition, optimization using the Taguchi method, which sets the reinforcement particle size as a noise factor, and regression analysis as a function of the reinforcement content, are new approaches that have not been reported before.

For this purpose, talc-reinforced PP and GF-reinforced PP were injection-molded under different conditions, and the differences in the shrinkage in the FD and TD, as well as shrinkage deviation between directions, were measured. On the basis of the experimental results, the optimal conditions for shrinkage prevention were identified through analysis of variance (ANOVA), the Taguchi method, and regression analysis. In particular, in this study, the particle size of the reinforcing agent was set as an uncontrollable factor (noise factor in the Taguchi method). This was expected to provide robust conditions for factors that are not precisely controlled, such as the particle size, in the actual injection-molding process. It was expected that this study would provide accurate guidelines for minimizing shrinkage in the injection molding of polymer composites.

## 2. Materials and Methods

### 2.1. Materials

PP supplied by Hanwha Total (Seoul, Korea) was used as the base material. Two types of talc (Hanwha Total, Seoul, Korea) were used as a reinforcing material. Talc is a thin plate-like reinforcing agent [22,23]. The average particle sizes (D50) of the two types of talc were 5.2 and 1.8 μm (denoted big and small, respectively). Each type had a specific gravity of 2.7. In addition, two types of GF (Owens Corning, Toledo, Ohio, America) were used as reinforcing materials. GF is an elongated thread-like reinforcing agent [24,25]. The average nominal diameters of the two types of GF were 13 and 10 μm (denoted big and small, respectively), and the chop lengths were both 4.3 mm. In addition, the specific gravities were both 2.6. In accordance with the reinforcement factors, the base material and the reinforcing agents were compounded, using a twin-screw extruder (TEK 25, SMPLATEK, Ansan, Korea) to produce talc-reinforced PP composites (PP/T) and glass-fiber-reinforced PP composites (PP/GF).

### 2.2. Sample Preparation

The test specimen for shrinkage was prepared in the shape of a thin rectangle of dimensions 150 mm × 100 mm × 1.8 mm. Test samples (parts) were injection molded with a 120-ton injection molding machine (WOOJIN SELEX-E120, Chungcheong, Korea). The injection temperature was 200 °C and the mold temperature was 40 °C. The injection pressure was 8 MPa. In addition, the holding pressure was 6.4 MPa (80% of the injection pressure) and the back pressure was 2 MPa. The injection speed was 40 mm/s, and the rotation speed of the injection molding screw was 60 rpm. The cooling time of the test samples was 40 s. More than seven samples were injection-molded, for each experimental condition.

### 2.3. Shrinkage Measurements

**Figure 1:**
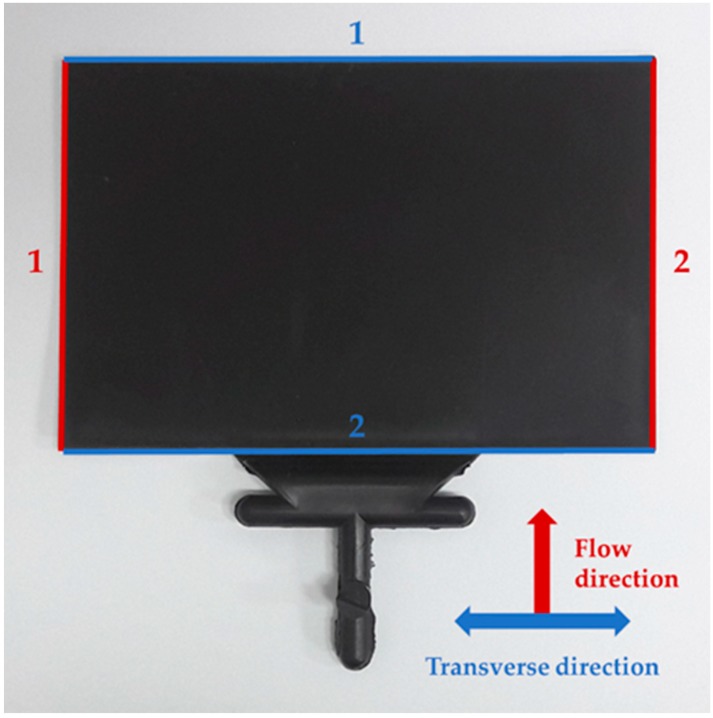
Shrinkage measurement positions of the test specimen.

Forty-eight hours after injection molding, the shrinkage was calculated by measuring the length of the test specimen. The lengths of seven samples were measured for each experimental condition, using Vernier calipers, and the maximum and minimum values were excluded. The shrinkage was calculated by measuring the lengths of five test samples at each condition, and the shrinkage was determined as follows:

(1) $$\mathrm{Shrinkage}\;(\%)=\frac{l_{\mathrm{mold}}-l_{\mathrm{part}}}{l_{\mathrm{mold}}}*100,$$

where $l_{\mathrm{mold}}$ represents the mold length and $l_{\mathrm{part}}$ represents the part length. Locations where shrinkage was measured and averaged on the test specimen are shown in Figure 1. There were two shrinkage measurement locations for the FD (red line in Figure 1) and TD (blue line in Figure 1). Differential shrinkage, which represents the deviation of the shrinkage between flow and transverse directions, was calculated using Equation (2).

(2) Differential shrinkage (%)=/Shrinkage in FD−Shrinkage in TD/

### 2.4. Distribution of Reinforcements

We confirmed the distribution of the reinforcements (talc and GF) in the base material, PP, using X-ray micro-computed tomography (Micro-CT). Using Micro-CT (Bruker Co., Billerical, Skyscan 1272, Massachusetts, United States), we captured cross-sectional images of the skin-core structure, to examine the distribution of the reinforcements and their orientation in PP.

### 2.5. Design of Experiments

The experiments were designed to find the optimal type and content of reinforcement that would minimize shrinkage. There were three factor types in the experiment. The type and content of the reinforcement were controllable factors, whereas the size of the reinforcement was set as an uncontrollable noise factor (Table 1). In this study, we conducted experiments on all conditions, in random order (Table 2). The shrinkage in the FD and TD, as well as the differential shrinkage, were compared at each condition.

### 2.6. Optimization Methods

From the experimental results, ANOVA, the Taguchi method, and regression analysis were applied to find the optimum conditions for preventing the shrinkage in the FD and TD, as well as the differential shrinkage.

ANOVA is a method to identify which factors are more influential among others. The dispersion of the characteristic values is expressed as a sum of squares and is divided by the number of degrees of freedom of each error, and the variance calculated by each factor is compared with the variance of the error [26].

The Taguchi method is a method for finding a robust optimal condition from the noise factors, which cannot be controlled by adjusting the level of the controllable factors. This robust condition can be calculated from the high signal-to-noise (S/N) ratio. A smaller shrinkage in the FD and TD, and a smaller differential shrinkage indicate better values; the corresponding S/N ratio equation is shown in Equation (3) [27].

(3) $$S/N\;\mathrm{ratio}\;(\mathrm{dB})\;=-10log_{10}\overset{n}{\underset{i=1}{\sum }}\frac{y_{i}^{2}}{n}$$

Here, *n* is the number of replications and *y* is the experimental value. 

Regression analysis is an analytical method that determines the correlation between several independent variables and dependent variables. A model showing the correlation between variables can be obtained by statistical methods and can be used to predict untested experimental conditions [28].

## 3. Results and Discussion

### 3.1. Effect of the Reinforcement on Shrinkage

**Figure 2:**
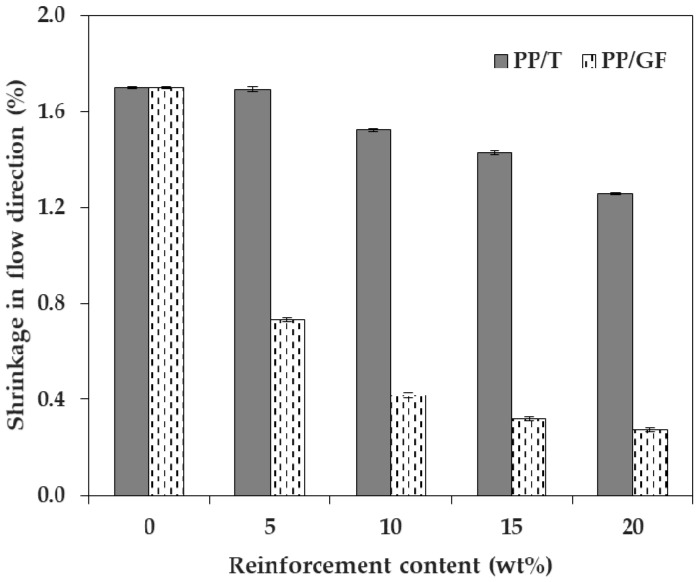
Shrinkage of talc-reinforced polypropylene composites (PP/T) and glass-fiber-reinforced polypropylene composites (PP/GF) in the flow direction, depending on the reinforcement type.

**Figure 3:**
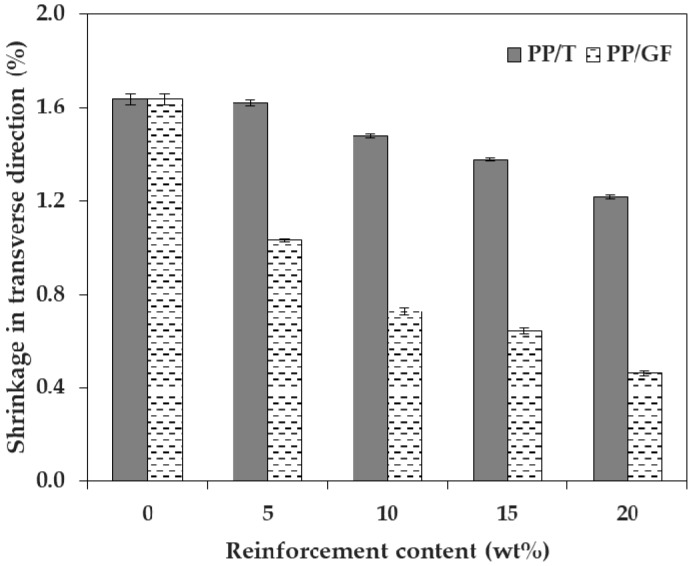
Shrinkage of PP/T and PP/GF in the transverse direction, depending on reinforcement type.

We measured and analyzed the shrinkage in three directions, upon the incorporation of talc and GF into PP, in which the reinforcement size factor was small. Figure 2 and Figure 3 show the shrinkage tendencies in FD and TD. The shrinkage was reduced with the addition of both talc and GF. The shrinkage of PP decreased in all directions, as the content of both talc and GF increased. Therefore, it was reasonable to add talc or GF, to reduce the shrinkage of PP. The reason for this was that both types of reinforcement had a lower coefficient of thermal expansion than the polymer matrix [10].

In particular, the addition of GF significantly reduced the shrinkage of PP in the two directions, compared with the addition of talc. The shrinkages of PP/T were 1.257% and 1.216% (FD and TD), and the shrinkages of PP/GF were 0.277% and 0.462% (FD and TD), at a reinforcement content of 20 wt %. It could be seen that the shrinkages of PP/T were about 4.54 times (FD) and 2.63 times (TD) higher than those of PP/GF. Therefore, the incorporation of GF into PP was more effective than the incorporation of talc, for minimizing shrinkage.

The shrinkage of PP/GF was reduced sharply at a GF content of 5 wt %, but the shrinkage of PP/T showed a relatively slight reduction at a talc content of 5 wt %. When the reinforcement content was 5 wt %, the shrinkage of PP/T decreased by 0.006% (FD) and 0.016% (TD), compared to that of PP alone, but the shrinkage of PP/GF decreased by 0.967% (FD) and 0.606% (TD), compared to that of PP. If the shrinkage of PP by the incorporation of a small amount of reinforcement (5 wt %) was required, GF was more useful than talc. This was expected because GF (5 × 10^−6^ 1/C) had a lower thermal expansion coefficient than talc (10^−5^ 1/C) [29].

**Figure 4:**
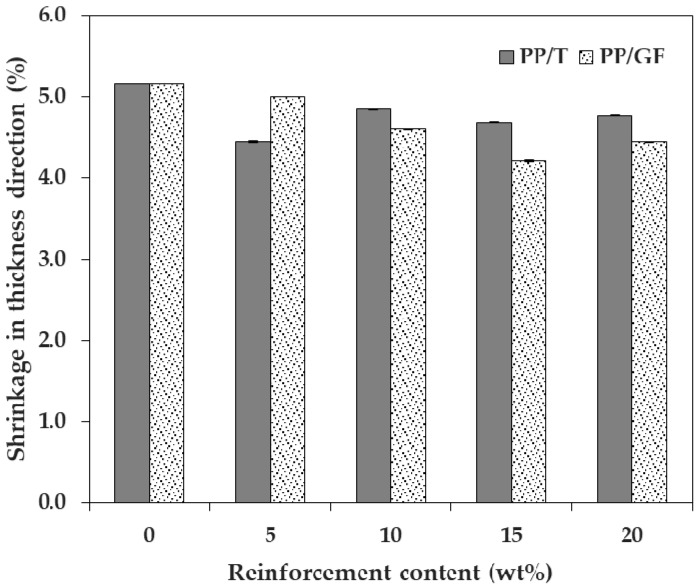
Shrinkage of PP/T and PP/GF in the thickness direction, depending on reinforcement type.

The shrinkage in the thickness direction decreased when the reinforcements were incorporated, as compared to when no reinforcement was added. However, no constant trend was observed with the increasing reinforcement content (Figure 4), indicating that the shrinkage in the thickness direction was not significantly affected by the reinforcement content. Since the reinforcements were oriented close to the FD, and the thickness was much smaller than the dimensions of the FD and TD, the minimization of shrinkage in the thickness direction, upon the addition of reinforcement, was insignificant. In addition, the shrinkage in the thickness direction (more than 4%) was much larger than the shrinkage in the FD or TD as a whole. This trend was similar to that observed in other studies on the shrinkage of PP [30,31].

**Figure 5:**
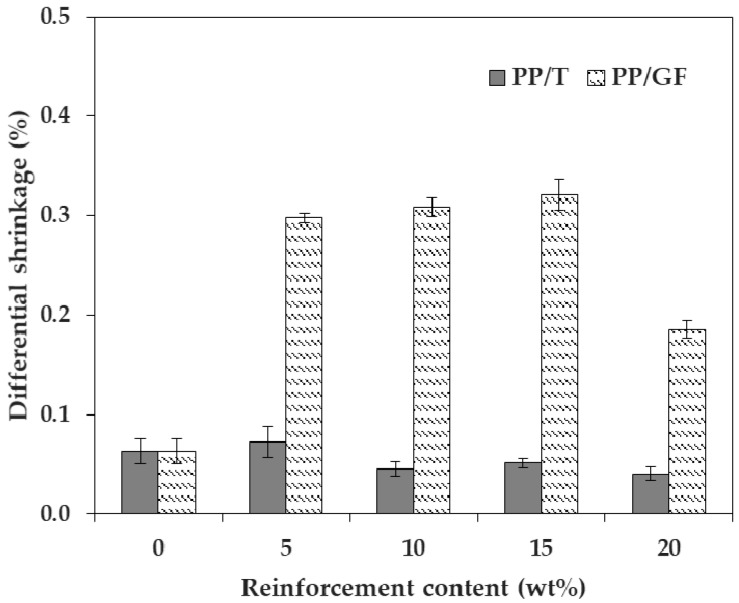
Differential shrinkage of PP/T and PP/GF, depending on the reinforcement type.

The differential shrinkage of PP/GF was about five times larger than that of PP/T, on average (Figure 5). When talc was incorporated, the differential shrinkage was similar to that of PP without reinforcement. The differential shrinkage of PP with no incorporated reinforcement was 0.063%, and the differential shrinkages of PP/T were from 0.041% (at 20 wt %) to 0.073% (at 5 wt %). The base material, PP, itself underwent anisotropic shrinkage (high differential shrinkage), and this could be improved by incorporating talc. PP is a semicrystalline polymer and is different from the amorphous polymers, which undergo isotropic shrinkage. Semicrystalline polymers show a sharp melting transition during melting and partial crystallization, during cooling [10,20]. As the crystallization progressed, forming a regular lattice chain, the volume of PP reduced sharply. Therefore, the shrinkage of PP became larger as the crystallized portions increased. On the other hand, when GF was incorporated, the differential shrinkage was much higher than that of PP without reinforcement. The differential shrinkages of PP/GF were 0.186% (at 20 wt %) to 0.321% (at 15 wt %).

**Figure 6:**
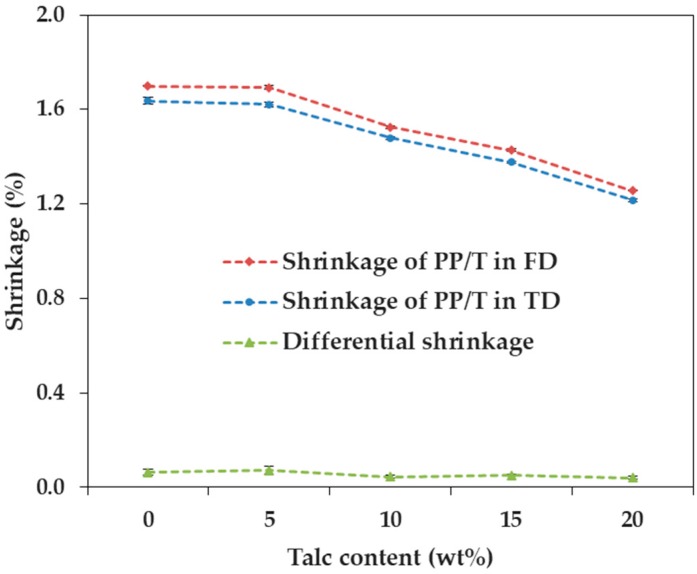
Shrinkage of PP/T as a function of talc content.

**Figure 7:**
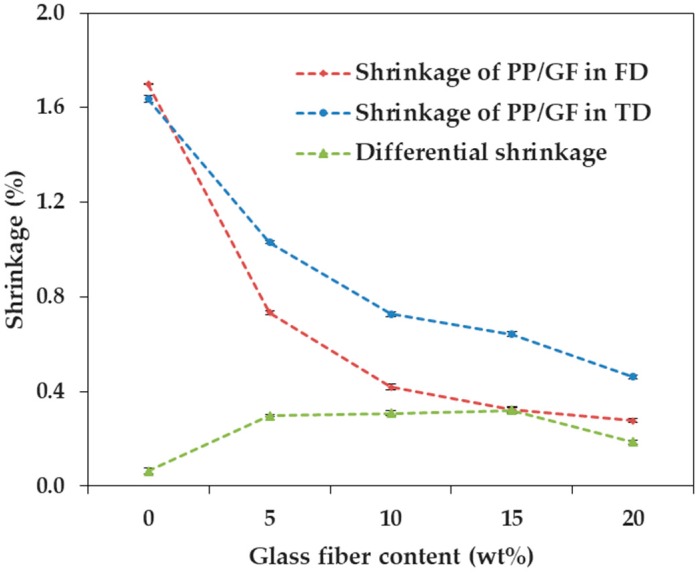
Shrinkage of PP/GF as a function of glass fiber content.

In other words, as the content of talc increased, the shrinkage of PP/T decreased, similarly, in both directions (Figure 6). The shrinkage of PP/T decreased by 0.111% (FD) and 0.105% (TD), on average, for every 5 wt % increase in talc content. On the other hand, the shrinkage of PP/GF in the FD, decreased more than that in the TD, as the GF contents increased (Figure 7). The shrinkage of PP/GF decreased by 0.356% (FD) and 0.294% (TD), on average, for every 5 wt % increase in GF content. 

**Figure 8:**
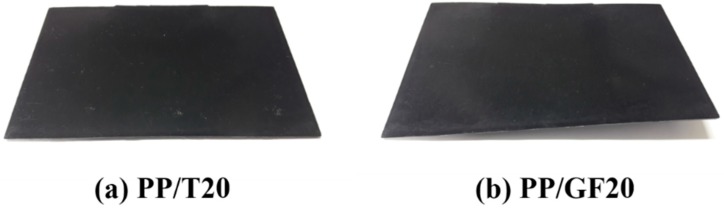
Warpage of test samples: (**a**) PP/T20 and (**b**) PP/GF20.

As the differential shrinkage increased, warpage of the injection molded parts occurred, so it was best to minimize the differential shrinkage. Compared with talc, GF minimized the shrinkage to a greater extent, but the effect on the differential shrinkage was rather worse. Therefore, considering warpage, talc was a better choice than GF (Figure 8). This was because the GF had a relatively higher aspect ratio than talc, which made it easier to align along the FD. The shrinkage in FD was greatly reduced if the orientation of GF was close to FD. However, since there was no remarkable reduction in shrinkage in the TD, a differential shrinkage occurred between the directions, and this worsened as the aspect ratio increased [32,33].

### 3.2. Shrinkage as a Function of Location

**Figure 9:**
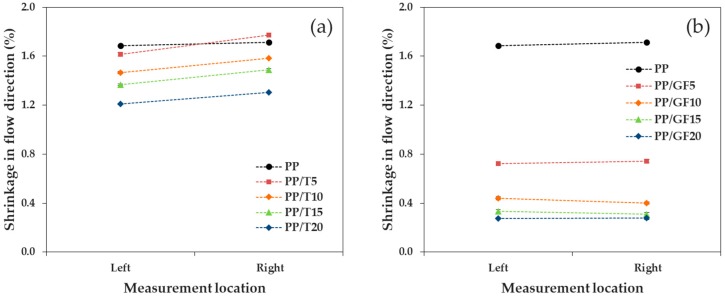
Shrinkage in the flow direction, as a function of the measurement location: (**a**) PP/T and (**b**) PP/GF.

**Figure 10:**
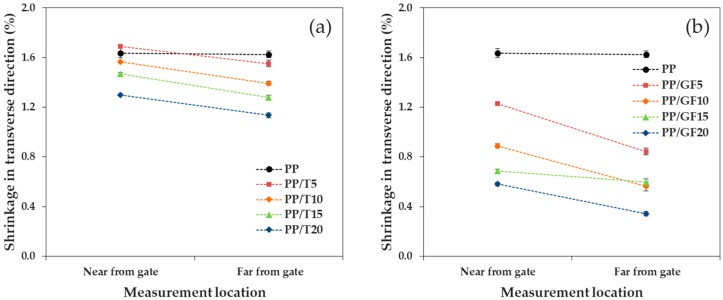
Shrinkage in the transverse direction, as a function of the measurement location: (**a**) PP/T and (**b**) PP/GF.

We confirmed the tendency of shrinkage as a function of the measurement location, when talc and GF were incorporated into PP, where the reinforcement size factor was small. As shown in Figure 9 and Figure 10, the shrinkages on the left and right sides of the FD and bottom (near the gate) and top (far from the gate) of the TD were compared (the geometric measurement locations are shown in Figure 1). As a whole, the addition of the reinforcement increased the shrinkage difference. In addition, the difference in shrinkage (by position) in the TD was larger than that in the FD. The shrinkage measurement positions in the FD were isotropic from the gate and exhibited a comparably similar reinforcement orientation. However, the shrinkage measurement positions in the TD were at different distances from the gate and exhibited a different reinforcement orientation.

In the FD, PP/T, and PP/GF showed a similar shrinkage in the left and right positions, but the shrinkage difference of PP/GF was smaller than that of PP/T. The average shrinkage difference of PP/T was 0.123%, while that of PP/GF was 0.022%. GF, with a high aspect ratio, was more uniformly orientated in the FD and exhibited a more uniform distribution, relative to the randomly oriented talc.

In the TD, PP/T showed a similar shrinkage on the bottom and top, although the shrinkage of PP/GF at the two positions were different. The average shrinkage difference of PP/T was 0.165%, while that of PP/GF was 0.259%. It could be seen that the shrinkage difference of PP/GF was larger than that of PP/T, which could be attributed to the orientation of GF.

### 3.3. Distribution of Reinforcement

**Figure 11:**
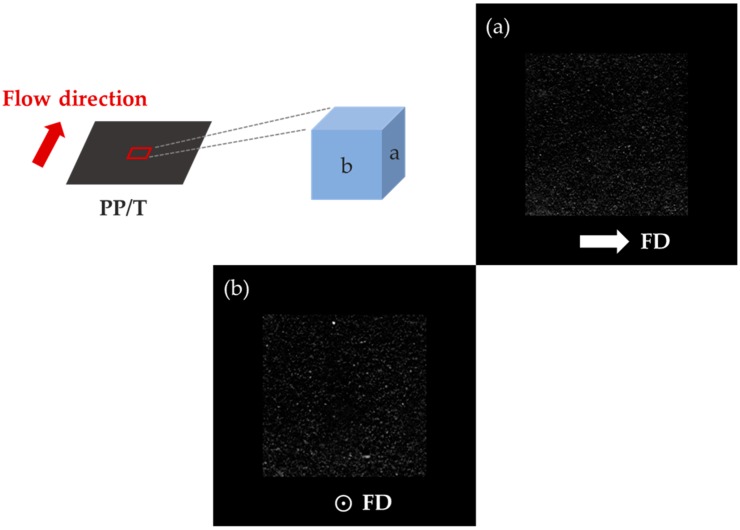
Distribution of talc in polypropylene (PP) measured by Micro-CT: (**a**) side and (**b**) front.

**Figure 12:**
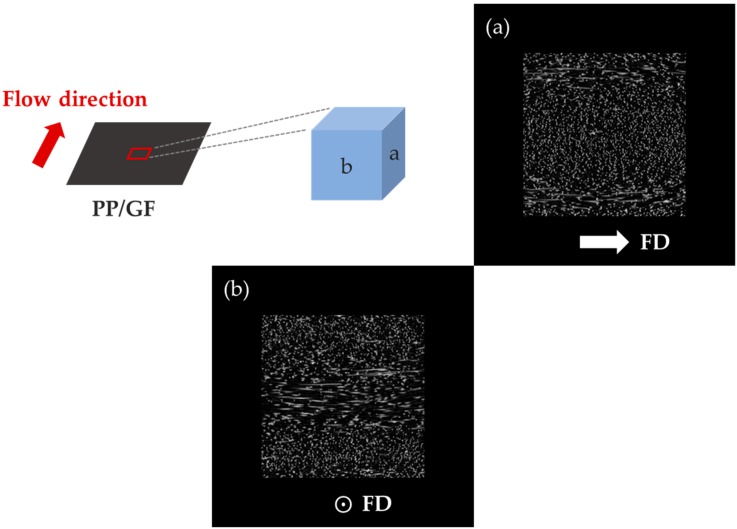
Distribution of glass fibers (GF) in PP measured by Micro-CT: (**a**) side and (**b**) front.

**Figure 13:**
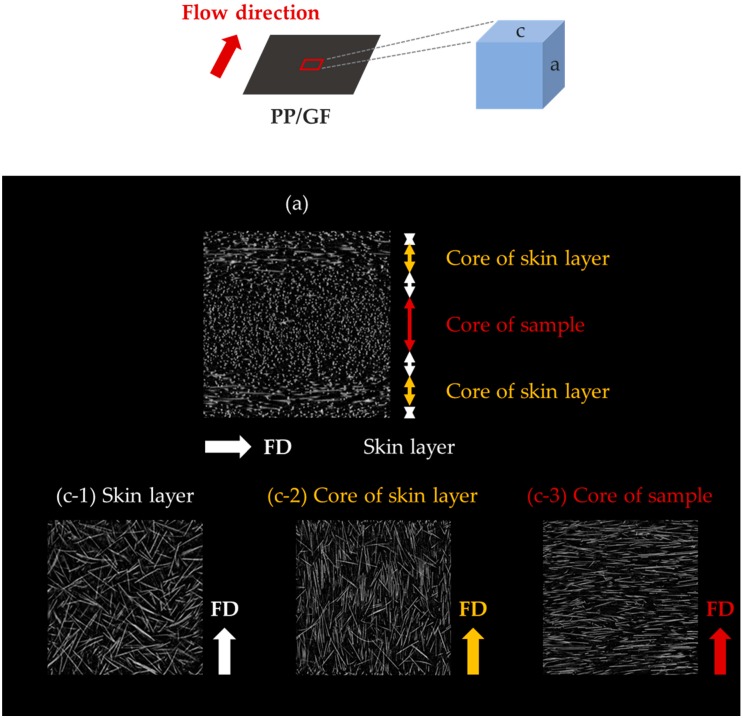
Orientation of glass fiber in PP in the different layers: (**a**) side and (**c**) top.

We have investigated the different shrinkage trends of PP/T and PP/GF that was caused by the reinforcement orientation. For this, we examined the reinforcement distributions by Micro-CT (Figure 11, Figure 12 and Figure 13). The reinforcement content was 20 wt %, and the size was small in this measurement.

It is clear from Figure 11 that all the talc in the cross-sections of PP/T were randomly distributed. There was no uniform distribution that appeared in the flow direction, transverse direction, and thickness direction.

On the other hand, GF in the cross-sections of PP/GF was very distinctly oriented (Figure 12). In particular, glass fibers exhibited different orientations in the skin layers near the surface of the sample and in the core inside it (Figure 13). The skin layers were divided into (i) skin layers without the core of the skin layers and (ii) a core skin layer. GF in the layers showed different orientations. They were randomly oriented in the skin layers, except in the core of the skin layers, where they were oriented parallel to the flow direction in the core of the skin layer. In the core, which is the central layer of the sample, they were oriented perpendicular to the flow direction. The orientation of GF was constant along each direction and the layers, and it was analyzed that this orientation caused a significant differential shrinkage between the FD and the TD. This fiber orientation in the injection mold was similar to that observed in other studies [34,35].

### 3.4. Analysis of Variance (ANOVA)

One-way ANOVA was performed for the three factors (reinforcement type, reinforcement content, and reinforcement size) and the three outcomes (shrinkages in the FD and TD, and the differential shrinkage) of the reinforcements. The number of replications was five. F-tests were conducted to determine whether the mean values for each level of the factor were the same or not. If the *p*-value of a factor was less than 0.05, the factor could be assumed to be significant.

Table 3 shows the ANOVA results for the shrinkage in the FD. In the case of the reinforcement factor, the *p*-value was 0, which was the most significant factor among the three. In addition, the content and size of reinforcement were not significant factors because the *p*-values were greater than 0.05. The ANOVA results for shrinkage in the TD showed that the reinforcement factor (*p*-value: 0) was the most significant factor (Table 4). The reinforcement content was the next most important factor (*p*-value: 0.005), and the reinforcement size was not a significant factor.

The ANOVA results for differential shrinkage are shown in Table 5. The *p*-value of the reinforcement factor was 0, making it the most significant factor for differential shrinkage. The other two factors were relatively insignificant (*p*-values: 0.290 and 0.567).

The analysis results for the three shrinkage values indicated that the type of reinforcement was the most significant factor (average *p*-value: 0). The next most significant factor was the reinforcement content, but the influence was not significant, compared to the effect of the reinforcement type. Finally, the reinforcement size was found to be an insignificant factor, overall. Therefore, to improve the shrinkage of PP, the factors should be considered in the order of reinforcement type and reinforcement content. The reinforcement size does not require consideration unless there is a large difference between the size of the particles.

Two-way ANOVA was performed to examine how the two variables (reinforcement type and reinforcement content) affected shrinkage and whether there was any interaction between these factors.

**Figure 14:**
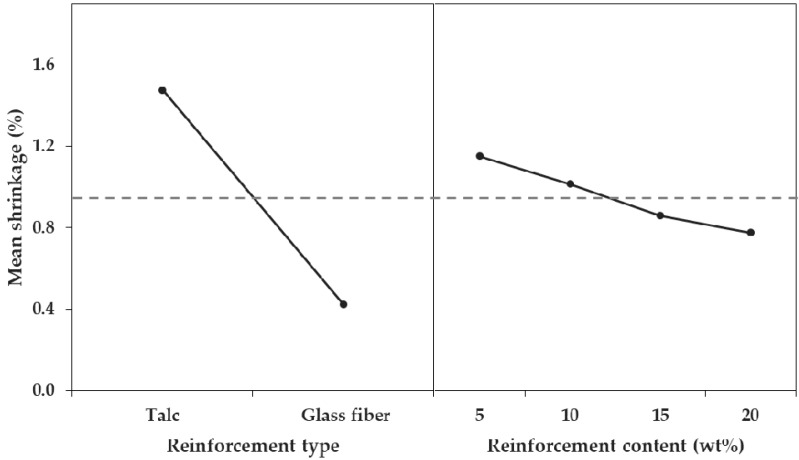
Main effect plot for shrinkage in the flow direction.

**Figure 15:**
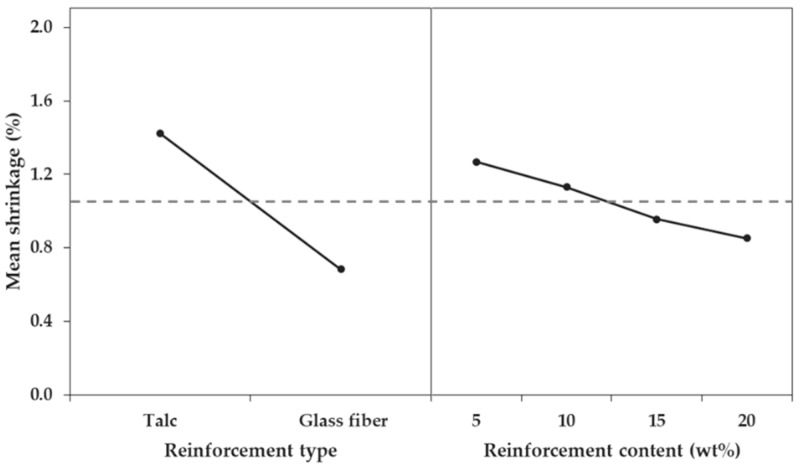
Main effect plot for shrinkage in the transverse direction.

First, the influence of the factors on the shrinkage in the FD and TD was analyzed, and the order or influence was reinforcement type, reinforcement content, and interaction between reinforcement type and reinforcement content. As the *p*-values of all three factors were less than 0.05, the factors were all significant for shrinkage in the FD and TD (Table 6 and Table 7). However, the *p*-values of the factors were all close to zero; thus, the importance of the factors could be identified by the difference in the value of the F-ratios. The influence of each of the three factors was very different, based on F-ratio values. The F-ratios of the reinforcement type factor were 9652.18% (FD) and 3827.91% (TD), those of the reinforcement content factor were 243.19% (FD) and 239.12% (TD), and those of the interaction between reinforcement type and reinforcement content were 5.64% (FD) and 8.13% (TD). This difference could be confirmed by comparing the main effects (Figure 14 and Figure 15).

**Figure 16:**
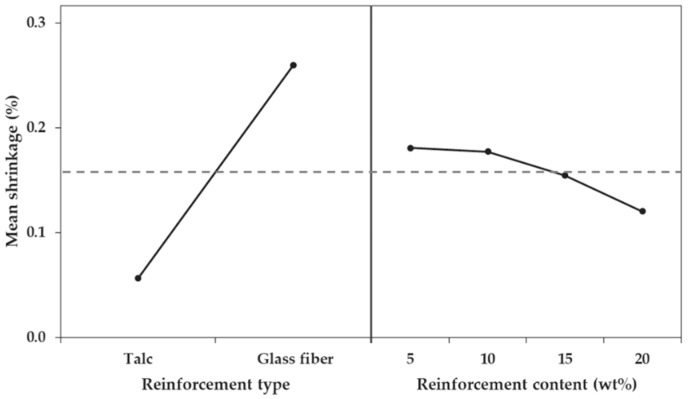
Main effect plot for differential shrinkage.

Next, the influence of the three factors on differential shrinkage was confirmed in the order of reinforcement type, reinforcement content, and the interaction between reinforcement type and reinforcement content. As the *p*-values of the three factors were less than 0.05, all factors were significant for differential shrinkage (Table 8). The F-ratio of each factor showed that influence of the reinforcement type was overwhelming, but the effect of the reinforcement content was relatively small, compared to the effect on the shrinkage in the FD and TD. The F-ratio of the reinforcement factor was 708.69%, that of the reinforcement content factor was 13.37%, and that of the interaction between the reinforcement type and reinforcement content was 6.10%. This meant that the variation in the differential shrinkage was not large and did not depend on the reinforcement content. Thus, differential shrinkage could not be controlled by varying the reinforcement content. This difference in influence could also be seen from the main effect comparison (Figure 16).

**Figure 17:**
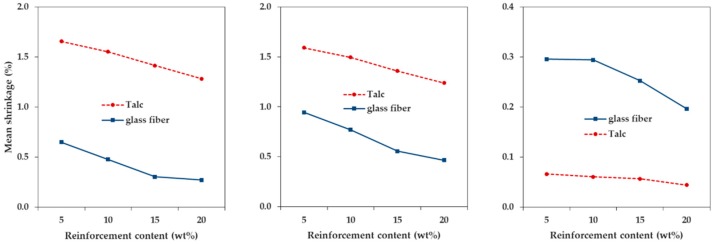
Interaction plot for the mean (**a**) shrinkage in the flow direction, (**b**) shrinkage in the transverse direction, and (**c**) differential shrinkage.

Finally, we examined the interaction between the reinforcement type and reinforcement content for the three outcomes. The magnitude of the interaction was readily apparent in the interaction plot (Figure 17). In the interaction plot, a small interaction is indicated by parallel lines, whereas intersecting lines indicate a large interaction [36]. The interaction plots were almost parallel, so the interaction between factors was low.

### 3.5. The Taguchi Method

The optimal conditions for the controllable factors for shrinkage were analyzed by the Taguchi method. The controllable factors were the reinforcement type (A) and the reinforcement content (B). The noise factor was the reinforcement size (N). The levels of the factors were 2 for A (talc and GF), 4 for B (5, 10, 15, and 20 wt %), and 2 for N (big and small size).

The optimum conditions were calculated, based on the condition that the S/N ratio was high. Table 9, Table 10 and Table 11 show all shrinkage values and S/N ratios for each noise factor. In the case of shrinkage in the FD and TD, it was found that the optimum condition was obtained when 20 wt % of GF was incorporated into the PP. The S/N ratios of shrinkage in FD and TD were the largest (11.358 dB (FD) and 6.621 dB (TD)) at condition number 8 with 20 wt % incorporated GF. On the other hand, differential shrinkage was found to be optimum when 20 wt % of talc was incorporated into the PP. The S/N ratio of differential shrinkage was the largest (26.755 dB) at condition number 4, with 20 wt % talc incorporated.

These results suggest that GF was a better reinforcing agent than talc, for the reduction of directional shrinkage, whereas talc was a better reinforcement than GF, for the reduction of differential shrinkage.

### 3.6. Regression Analysis

According to the above analysis, if we want to minimize shrinkage in the FD and TD, we can incorporate as much GF as possible, and, if we want to minimize differential shrinkage, we can incorporate as much talc as possible. However, if we want to minimize both directional shrinkages and the differential shrinkage, both reinforcing agents must be added.

**Figure 18:**
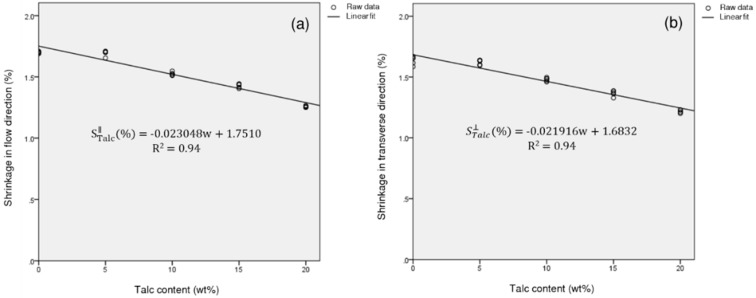
Regression analysis plot for the shrinkage of PP/T: (**a**) Shrinkage in the flow direction, and (**b**) shrinkage in the transverse direction.

**Figure 19:**
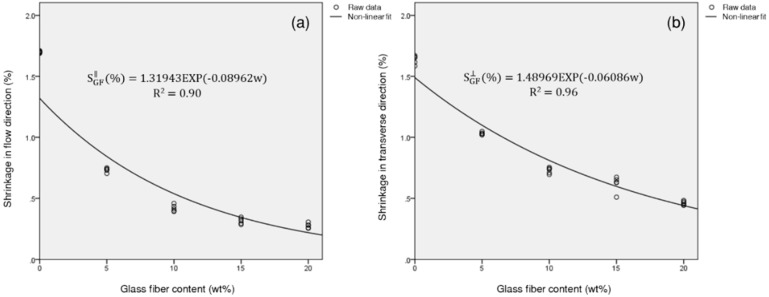
Regression analysis plot for the shrinkage of PP/GF: (**a**) Shrinkage in the flow direction, and (**b**) shrinkage in the transverse direction.

To find the optimum mixed reinforcing conditions, regression analysis was performed with the reinforcement content as a variable. For the regression analysis, only the small size reinforcing agents were used. Regression equations for the shrinkage of PP/T in the FD and TD and those of PP/GF in the FD and TD were calculated as a function of the reinforcement content. The experimental values of PP/T showed a good fit with the linear regression model, and the experimental values of PP/GF showed a high fitness with the exponential non-linear regression model (Figure 18 and Figure 19). The regression equation for the PP composite, reinforced with talc and GF, was as follows:

(4) $$S^{∥}=\frac{w_{talc}}{w_{total}}S_{talc}^{∥}+\frac{w_{GF}}{w_{total}}S_{GF}^{∥},$$

(5) $$S^{⊥}=\frac{w_{talc}}{w_{total}}S_{talc}^{⊥}+\frac{w_{GF}}{w_{total}}S_{GF}^{⊥}.$$

where $S^{∥}$ is the total shrinkage in the FD, and $S^{⊥}$ is the total shrinkage in the FD. $S_{talc}^{∥}$ and $S_{talc}^{⊥}$ are the shrinkage regression equations for PP/T in the FD and TD. $S_{GF}^{∥}$ and $S_{GF}^{⊥}$ are the shrinkage regression equations for PP/GF in the FD and TD. In addition, $w_{talc}$ and $w_{GF}$ represent the content of incorporated talc and GF, and $w_{total}$ is the total reinforcement content ($w_{total}>0)$.

In industry, there are restrictions on the optimal conditions for various factors. For example, in a case where the reinforcement content is limited to 20 wt %, the shrinkage in the FD and TD should be 1.2 % or less, and the differential shrinkage should be 0.1 % or less, the incorporation of one kind of reinforcement, such as talc or GF, will be unsatisfactory. However, the addition of 11 wt % of talc and 9 wt % of GF, can satisfy the required condition (Table 12). As another example, if the shrinkage in the FD and TD was 1% or less, the differential shrinkage should have been 0.15% or less, and the total reinforcing agent content should not have exceeded 20 wt %; again, this condition could not be achieved with a single reinforcing agent. However, the addition of 7 wt % of talc and 13 wt % of GF into the PP, could achieve this value. In this way, we could predict optimal conditions for various dependent variables, using the regression model.

## 4. Conclusions

In this study, we investigated the optimal reinforcement factors to minimize the shrinkage of talc- and GF-reinforced PP composites, after injection molding. The reinforcement factors were reinforcement type (talc and GF), reinforcement content (0, 5, 10, 15, and 20 wt %), and reinforcement size (big and small). The type and content of the reinforcement were controllable variables, and reinforcement size was set as an uncontrollable variable. The samples for all experimental conditions were injection-molded to measure the differential shrinkage, as well as the shrinkages in the flow direction, transverse direction, and thickness direction. In addition, the shrinkage of PP/T and PP/GF, at different measurement locations and the distribution of reinforcements in the samples, were confirmed. For the measured shrinkage results, optimal conditions were obtained by applying ANOVA, the Taguchi method, and regression analysis. It was confirmed that the reinforcement type was the most influential factor of reinforcement type, reinforcement content, and reinforcement size. Of these factors, the reinforcement size was found to be relatively insignificant, compared to the other factors. Therefore, when a reinforcing agent was incorporated into PP to minimize the shrinkage of PP, the factors should be considered in order of reinforcement type, reinforcement content, and reinforcement size. The optimum condition with minimum shrinkage in the FD and TD for the noise factor were found to be 20 wt % of GF, and the optimal condition with minimum differential shrinkage was found to be 20 wt % of talc. Finally, regression analysis models were prepared to identify the conditions where the shrinkage in the FD and TD, as well as the differential shrinkage, satisfy specified values, simultaneously. It is expected that this study will be helpful for understanding the optimum reinforcement factors to minimize the shrinkage of PP composites.

## Figures and Tables

**Table 1:** Factors and levels of the experiments.

Level	Controllable Factor		Noise Factor
	Reinforcement Type	Reinforcement Content (wt %)	Reinforcement Size
1	Talc	5	Big
2	Glass fiber	10	Small
3		15	
4		20	

**Table 2:** Experimental conditions.

Number	Reinforcement Type	Reinforcement Content (wt %)	Reinforcement Size
1	-	0	-
2	Talc	5	Big
3	Talc	5	Small
4	Talc	10	Big
5	Talc	10	Small
6	Talc	15	Big
7	Talc	15	Small
8	Talc	20	Big
9	Talc	20	Small
10	Glass fiber	5	Big
11	Glass fiber	5	Small
12	Glass fiber	10	Big
13	Glass fiber	10	Small
14	Glass fiber	15	Big
15	Glass fiber	15	Small
16	Glass fiber	20	Big
17	Glass fiber	20	Small

**Table 3:** One-way ANOVA of shrinkage in the flow direction.

Factor	Degrees of Freedom	Sum of Squares	Mean Square	F-Ratio (%)	*p*-Value
Reinforcement type	1	22.197	22.197	919.82	0.000
Reinforcement content	3	1.678	0.5593	1.90	0.137
Reinforcement size	1	0.0026	0.0026	0.01	0.927

**Table 4:** One-way ANOVA of shrinkage in the transverse direction.

Factor	Degrees of Freedom	Sum of Squares	Mean Square	F-Ratio (%)	*p*-Value
Reinforcement type	1	10.866	10.866	366.91	0.000
Reinforcement content	3	2.036	0.6788	4.63	0.005
Reinforcement size	1	0.0237	0.0237	0.14	0.709

**Table 5:** One-way ANOVA of differential shrinkage.

Factor	Degrees of Freedom	Sum of Squares	Mean Square	F-Ratio (%)	*p*-Value
Reinforcement type	1	0.8248	0.8248	423.83	0.000
Reinforcement content	3	0.04670	0.01557	1.27	0.290
Reinforcement size	1	0.004117	0.004117	0.33	0.567

**Table 6:** Two-way ANOVA of shrinkage in the flow direction.

Factor	Degrees of Freedom	Sum of Squares	Mean Square	F-Ratio (%)	*p*-Value
Reinforcement type	1	22.197	22.197	9652.18	0.000
Reinforcement content	3	1.6778	0.5593	243.19	0.000
Reinforcement typeⅹcontent	3	0.0389	0.0130	5.64	0.002

**Table 7:** Two-way ANOVA for shrinkage in the transverse direction.

Factor	Degrees of Freedom	Sum of Squares	Mean Square	F-Ratio (%)	*p*-Value
Reinforcement type	1	10.866	10.866	3827.91	0.000
Reinforcement content	3	2.0363	0.6788	239.12	0.000
Reinforcement typeⅹcontent	3	0.0693	0.0231	8.13	0.000

**Table 8:** Two-way ANOVA of differential shrinkage.

Factor	Degrees of Freedom	Sum of Squares	Mean Square	F-Ratio (%)	*p*-Value
Reinforcement type	1	0.8249	0.8249	708.69	0.000
Reinforcement content	3	0.0467	0.01557	13.37	0.000
Reinforcement typeⅹcontent	3	0.0213	0.0071	6.10	0.001

**Table 9:** Signal-to-noise (S/N) ratio of shrinkage in the flow direction.

No.	A	B (wt %)	Shrinkage (%)						S/N Ratio (dB)
1	Talc	5	N1	1.664	1.618	1.644	1.587	1.598	−4.394
			N2	1.705	1.700	1.654	1.700	1.710	
2	Talc	10	N1	1.592	1.577	1.577	1.582	1.592	−3.835
			N2	1.526	1.516	1.526	1.547	1.511	
3	Talc	15	N1	1.414	1.373	1.419	1.414	1.399	−3.021
			N2	1.419	1.444	1.434	1.439	1.404	
4	Talc	20	N1	1.296	1.317	1.307	1.296	1.327	−2.164
			N2	1.251	1.256	1.251	1.261	1.266	
5	GF	5	N1	0.531	0.531	0.618	0.541	0.597	3.684
			N2	0.740	0.730	0.750	0.740	0.704	
6	GF	10	N1	0.541	0.500	0.556	0.531	0.536	6.374
			N2	0.434	0.393	0.398	0.413	0.459	
7	GF	15	N1	0.286	0.265	0.286	0.311	0.271	10.351
			N2	0.316	0.316	0.296	0.332	0.347	
8	GF	20	N1	0.271	0.276	0.260	0.260	0.250	11.358
			N2	0.260	0.306	0.281	0.281	0.255	

**Table 10:** S/N ratio of shrinkage in the transverse direction.

No.	A	B (wt %)	Shrinkage (%)						S/N Ratio (dB)
1	Talc	5	N1	1.595	1.551	1.572	1.545	1.551	−4.041
			N2	1.602	1.595	1.636	1.639	1.633	
2	Talc	10	N1	1.511	1.507	1.507	1.507	1.511	−3.489
			N2	1.470	1.460	1.497	1.487	1.484	
3	Talc	15	N1	1.328	1.328	1.345	1.355	1.352	−2.668
			N2	1.379	1.386	1.372	1.386	1.362	
4	Talc	20	N1	1.264	1.254	1.264	1.261	1.264	−1.861
			N2	1.210	1.213	1.230	1.200	1.227	
5	GF	5	N1	0.848	0.852	0.865	0.845	0.875	0.463
			N2	1.034	1.021	1.048	1.031	1.021	
6	GF	10	N1	0.821	0.818	0.804	0.794	0.825	2.252
			N2	0.737	0.696	0.710	0.754	0.744	
7	GF	15	N1	0.510	0.453	0.470	0.446	0.460	4.997
			N2	0.629	0.632	0.673	0.652	0.629	
8	GF	20	N1	0.466	0.466	0.480	0.470	0.470	6.621
			N2	0.450	0.483	0.463	0.443	0.473	

**Table 11:** S/N ratio of differential shrinkage.

No.	A	B (wt %)	Shrinkage (%)						S/N Ratio (dB)
1	Talc	5	N1	0.069	0.067	0.072	0.043	0.046	23.038
			N2	0.103	0.104	0.018	0.060	0.077	
2	Talc	10	N1	0.082	0.070	0.070	0.075	0.082	23.958
			N2	0.056	0.056	0.029	0.059	0.027	
3	Talc	15	N1	0.086	0.045	0.074	0.058	0.047	24.676
			N2	0.040	0.059	0.062	0.054	0.042	
4	Talc	20	N1	0.032	0.063	0.043	0.036	0.063	26.755
			N2	0.040	0.042	0.020	0.061	0.039	
5	GF	5	N1	0.318	0.321	0.248	0.304	0.278	10.559
			N2	0.294	0.291	0.297	0.291	0.316	
6	GF	10	N1	0.280	0.318	0.248	0.263	0.289	10.600
			N2	0.303	0.303	0.312	0.340	0.284	
7	GF	15	N1	0.225	0.188	0.184	0.135	0.189	11.576
			N2	0.312	0.316	0.377	0.321	0.282	
8	GF	20	N1	0.196	0.191	0.220	0.210	0.220	14.092
			N2	0.189	0.177	0.182	0.162	0.218	

**Table 12:** Shrinkage prediction by regression analysis.

No.	Talc (wt %)	GF (wt %)	Shrinkage in FD (%)	Shrinkage in TD (%)	Differential Shrinkage (%)
1	20	0	1.290	1.245	0.045
2	19	1	1.308	1.274	0.034
3	18	2	1.313	1.292	0.021
4	17	3	1.307	1.300	0.006
5	16	4	1.290	1.300	0.009
6	15	5	1.265	1.291	0.026
7	14	6	1.231	1.274	0.043
8	13	7	1.190	1.249	0.059
9	12	8	1.142	1.218	0.076
10	11	9	1.089	1.181	0.092
11	10	10	1.030	1.137	0.108
12	9	11	0.965	1.088	0.123
13	8	12	0.897	1.034	0.137
14	7	13	0.824	0.974	0.150
15	6	14	0.747	0.910	0.163
16	5	15	0.667	0.842	0.175
17	4	16	0.583	0.769	0.186
18	3	17	0.497	0.693	0.196
19	2	18	0.407	0.612	0.205
20	1	19	0.315	0.528	0.214
21	0	20	0.220	0.441	0.221
